# Antigenic Differences between AS03 Adjuvanted Influenza A (H1N1) Pandemic Vaccines: Implications for Pandemrix-Associated Narcolepsy Risk

**DOI:** 10.1371/journal.pone.0114361

**Published:** 2014-12-15

**Authors:** Outi Vaarala, Arja Vuorela, Markku Partinen, Marc Baumann, Tobias L. Freitag, Seppo Meri, Päivi Saavalainen, Matti Jauhiainen, Rabah Soliymani, Turkka Kirjavainen, Päivi Olsen, Outi Saarenpää-Heikkilä, Juha Rouvinen, Merja Roivainen, Hanna Nohynek, Jukka Jokinen, Ilkka Julkunen, Terhi Kilpi

**Affiliations:** 1 Department of Vaccinations and Immune Protection, National Institute for Health and Welfare, Helsinki, Finland; 2 Helsinki Sleep Clinic, Vitalmed Research Centre Helsinki and Haartman Institute, University of Helsinki, Helsinki, Finland; 3 Meilahti Clinical Proteomics Core Facility, Institute of Biomedicine/Biochemistry and Developmental Biology, and NeuroMed Research Program, University of Helsinki, Helsinki, Finland; 4 Department of Bacteriology and Immunology, Haartman Institute, University of Helsinki, Helsinki, Finland and Research Program Unit, Immunobiology, University of Helsinki, Helsinki, Finland; 5 National Institute for Health and Welfare, Public Health Genomics Research Unit, Biomedicum, Helsinki, Finland; 6 Department of Pediatrics, Children’s Hospital, Helsinki University Hospital, Helsinki, Finland; 7 Department of Child Neurology, Oulu University Hospital, Oulu, Finland; 8 Department of Pediatrics, Tampere University Hospital, Tampere, Finland; 9 Department of Chemistry and Biocenter Kuopio, University of Eastern Finland, Joensuu, Finland; 10 Department of Infectious Disease Surveillance and Control, National Institute for Health and Welfare, Helsinki, Finland; 11 Department of Virology, University of Turku, Turku, Finland; Federal University of São Paulo, Brazil

## Abstract

**Background:**

Narcolepsy results from immune-mediated destruction of hypocretin secreting neurons in hypothalamus, however the triggers and disease mechanisms are poorly understood. Vaccine-attributable risk of narcolepsy reported so far with the AS03 adjuvanted H1N1 vaccination Pandemrix has been manifold compared to the AS03 adjuvanted Arepanrix, which contained differently produced H1N1 viral antigen preparation. Hence, antigenic differences and antibody response to these vaccines were investigated.

**Methods and Findings:**

Increased circulating IgG-antibody levels to Pandemrix H1N1 antigen were found in 47 children with Pandemrix-associated narcolepsy when compared to 57 healthy children vaccinated with Pandemrix. H1N1 antigen of Arepanrix inhibited poorly these antibodies indicating antigenic difference between Arepanrix and Pandemrix. High-resolution gel electrophoresis quantitation and mass spectrometry identification analyses revealed higher amounts of structurally altered viral nucleoprotein (NP) in Pandemrix. Increased antibody levels to hemagglutinin (HA) and NP, particularly to detergent treated NP, was seen in narcolepsy. Higher levels of antibodies to NP were found in children with DQB1*06∶02 risk allele and in DQB1*06∶02 transgenic mice immunized with Pandemrix when compared to controls.

**Conclusions:**

This work identified 1) higher amounts of structurally altered viral NP in Pandemrix than in Arepanrix, 2) detergent-induced antigenic changes of viral NP, that are recognized by antibodies from children with narcolepsy, and 3) increased antibody response to NP in association of DQB1*06∶02 risk allele of narcolepsy. These findings provide a link between Pandemrix and narcolepsy. Although detailed mechanisms of Pandemrix in narcolepsy remain elusive, our results move the focus from adjuvant(s) onto the H1N1 viral proteins.

## Introduction

According to the new International Classification of Sleep Disorders (ICSD-3) narcolepsy is divided into type 1 and type 2 narcolepsy. Type 1 narcolepsy results from an immune-mediated destruction of hypocretin secreting neurons in hypothalamus [1]. It is characterized by excessive daytime sleepiness (EDS), cataplexy and often disturbed nocturnal sleep. We recently reported an increased risk of narcolepsy in association with an AS03 adjuvanted influenza A(H1N1) vaccine in Finnish children and adolescents following the nationwide vaccination campaign carried out with the Pandemrix vaccine during fall 2009 [2]. The risk of narcolepsy was more than 10-fold among vaccinated when compared to unvaccinated children and adolescents aged 4–19 years in 2009–2010. The association of Pandemrix vaccination and narcolepsy in children and adolescents has also been reported in Sweden, Norway, Ireland, France, and U.K. [3], [4], [5], [6], [7]. In adults, the Pandemrix vaccination has been associated with narcolepsy in France, Sweden and Finland [6], [8], [9].

In the general population, incidence of narcolepsy has been shown to be strongly associated with the HLA DQB1*0602 allele and more weakly associated with other genes regulating the function of immune cells [10], [11], [12], [13]. These genetic studies suggest that CD4+ T-lymphocytes play a role in the pathogenesis of narcolepsy and support the biological plausibility of vaccinations as an environmental trigger of narcolepsy based on their immunomodulatory effects. In earlier epidemiological and seroepidemiological research, streptococcal infections have been proposed as triggers of narcolepsy [14], [15]. Also some epidemiological observations suggest a role of H1N1 influenza infection in the development of narcolepsy [16], [17].

The association of the Pandemrix vaccine with narcolepsy suggests that the immune-mediated mechanisms leading to narcolepsy are activated by the AS03 adjuvanted H1N1 vaccine. The possible role vaccines, particularly those with adjuvants, may play in the triggering of autoimmune diseases has been previously discussed. To date, no comparative epidemiological study has found support for this hypothesis, except for the recent case of Pandemrix-associated narcolepsy [2], [3], [4], [5], [6], [7], [8], [9]. Adjuvants may cause non-specific activation of immune cells, and thus by-stander activation of narcolepsy related immunity could explain the increased risk of narcolepsy following vaccination. However, the role of adjuvants as a trigger of narcolepsy is far from certain. Indeed, no evidence exist on the association between narcolepsy and MF59 adjuvanted H1N1 vaccine or the AS03 adjuvanted H1N1 vaccine Arepanrix, which both contain squalene based adjuvant.

A recent report exploring T-cell reactivity against hypocretin in narcolepsy [18] identified H1N1 virus derived hemagglutinin (HA) peptides and hypocretin peptides that bind to HLA DQB1*06∶02 risk allele of narcolepsy and could thus be functional T-cell epitopes. T-cell reactivity to peptides of hypocretin or the crossreactivity with HA epitopes was not, however, reproducible in the later studies, and the report was retracted in July 2014. The role of HA, the major immunogen in influenza vaccines, as a trigger of narcolepsy is challenged also by the epidemiological observations. The risk of narcolepsy differs between Pandemrix and Arepanrix although both contain similar dose of HA and AS03 adjuvant, and the induction of HA specific antibodies has been reported to be equivalent according to the market authorization holder [19]. Furthermore, the dose of HA is actually four times higher in seasonal influenza vaccines than in adjuvanted H1N1 vaccines. While a number of other vaccine preparations were used on a large scale during H1N1 pandemic period and later, the available epidemiological data clearly shows that H1N1 vaccines other than Pandemrix do not confer the same high risk of narcolepsy.

Accordingly, the risk of narcolepsy conferred by AS03 adjuvanted Pandemrix can be interpreted to be related to some specific characteristics of Pandemrix. It is therefore interesting that the H1N1 antigen was produced according to different manufacturing processes for Arepanrix and Pandemrix, the two AS03 adjuvanted vaccines [20], [21], which may results in antigenic differences as we have earlier suggested [22].

In order to understand the mechanisms behind Pandemrix triggered narcolepsy, we explored the antigenic nature of Pandemrix and Arepanrix. We first evaluated the antigenic properties of Pandemrix and Arepanrix by studying the characteristics of humoral immune response against these two AS03 adjuvanted H1N1 vaccines in children who developed narcolepsy after Pandemrix vaccination and healthy children. We found enhanced levels of IgG-antibodies to a Pandemrix H1N1 viral antigen in the children with narcolepsy. The IgG-antibody reactivity in vaccinated individuals revealed differences in the viral antigenicity of Pandemrix when compared to Arepanrix. High-resolution gel electrophoresis, mass spectrometry analyses and Western-blot analyses showed higher amounts and particularly multimeric forms of viral nucleoprotein (NP) in Pandemrix. Furthermore, detergents used in the manufacturing of the Pandemrix H1N1 antigen suspension modified *in vitro* the antigenic epitopes of viral proteins, and enhanced binding of IgG-antibodies to Pandemrix detergent treated HA and NP were seen in the children with narcolepsy when compared to vaccinated healthy children, suggesting that these epitopes provide a link between narcolepsy and Pandemrix. Interestingly, children with DQB1* 06∶02 risk allele had elevated levels of antibodies to NP. Mice transgenic mice for DQB1* 06∶02 showed higher antibody response to NP than DQB1* 03∶02 transgenic mice when immunized with Pandemrix. Although a detailed mechanism of Pandemrix as a trigger of narcolepsy is not revealed, our results move the focus from adjuvant(s) or from other H1N1 virus vaccinations than Pandemrix onto the H1N1 viral proteins. These results indicate antigenic differences between the Pandemrix and Arepanrix vaccines that could be of importance in the development of narcolepsy in association with only Pandemrix vaccine.

## Study Subjects and Methods

### Ethical permissions

The children with narcolepsy were recruited to the immunological narcolepsy study at the neurological outpatient clinics in Finnish hospitals. Healthy siblings of children with type 1 diabetes were recruited at the Finnish Diabetes Registry during the same time period after Pandemrix vaccination campaign. The study protocol was approved by the ethics committee of the Helsinki University Central Hospital, and written informed consent was given by the children and their families.

The protocol of the studies in HLA-DQB1*06∶02 or HLA-DQB1*03∶02 transgenic mice was approved by the ethics committee, the Animal Experiment Board of the Regional State Administrative Agency for Southern Finland (ESAVI/1064/04.10.03/2012).

### Study subjects

47 children with narcolepsy diagnosed after Pandemrix vaccination were recruited to the immunological narcolepsy study at the neurological outpatient clinics in Finnish hospitals. All children were vaccinated in the end of 2009, developed first symptoms of narcolepsy after Pandemrix vaccination, and had type 1 narcolepsy. The blood samples were collected during 2011. As control children, 57 healthy siblings of children with type 1 diabetes were recruited at the Finnish Diabetes Registry during the same time period after Pandemrix vaccination campaign. Characteristics of the children with narcolepsy and healthy children are shown in table 1. Heparin whole blood was drawn and used for the HLA genotyping. Plasma samples were separated for the studies of humoral immune responses.

**Table 1:** Characteristics of children with narcolepsy and healthy children studied.

	Children with narcolepsy	Healthy children
Number of subjects	47	57
Vaccinated with Pandemrix (%)	100	100
Age at vaccination, median years (range)	11,7 (4,5–16,6)	7,5 (0,9–14,8)
Age at vaccination, mean years	11,5	8,3
Time between sampling and vaccination, median days	550	516
HLA DQB1*06∶02 genotype, N (%)	47 (100%)	20 (35%)
Gender, female, N (%)	28 (60%)	30 (53%)
Excessive daytime sleepiness (%)	100	NA
Cataplexy (%)	100	NA

### Methods

#### Animal experiments

HLA-DQB1*06∶02 or HLA-DQB1*03∶02 transgenic Ab0 NOD mice were purchased from Jackson Laboratory, Bar Harbor, Maine, and maintained under specific pathogen-free conditions [23]. Sex-matched groups (n = 10–12) of treatment-naïve male and female mice, 6 weeks old, were immunized with 50 µl Pandemrix into the right thigh muscle, vs. PBS control, and received boosts with the same dose on weeks 2, 4, and 8. Serum was obtained after 11 weeks by retroorbital bleeding under anesthesia (Ketamine/Xylazine), followed by asphyxiation with CO_2_. Approval for all procedures had been obtained from the animal research board of the Southern Finnish State Administrative Agency (ESAVI/1064/04.10.03/2012).

#### Enzyme linked solid-phase immunoassay (EIA) for IgG-antibodies binding to H1N1 antigen suspension

We used H1N1 antigen suspension of Pandemrix (GSK, Dresden, Germany) and Arepanrix (GSK, Quebec, Canada) vaccines as antigens, and the differences in the composition of the H1N1 antigens of Pandemrix and Arepanrix according to the product leaflets [20], [21] are shown in table 2.

**Table 2:** The differences in the composition of Pandemrix and Arepanrix H1N1 antigen suspension according to the product leaflets (references 20,21).

	Excipient	Original concentration
H1N1 antigen suspension of Pandemrix	Polysorbate 80 and Octoxynol 10 (Triton X-100)	not known
H1N1 antigen suspension of Arepanrix	Sodium deoxycholate	“trace amounts”

For the EIA, polystyrene plates (Nunc, Denmark) were coated with H1N1 antigen suspension (1∶10 dilution in phosphate buffered saline, PBS) and kept overnight at +4°C. As a control antigen, tetanus toxoid (coated at concentration of 46 µg/ml in PBS) was used. Plates were post-coated with 1% fetal bovine serum (FBS from Gibco) and incubated for 60 min at room temperature (RT), and then washed with PBS. Plasma samples diluted 1∶100 in 1% FBS were incubated as duplicates for two hours at RT. After washing with PBS, alkaline phosphatase conjugated anti- human IgG (Jackson ImmunoResearch, USA) was added (1∶3000 in 1% FBS for one hour at RT). P-nitrophenyl phosphate was used as substrate and color reaction read at 402 nm after 30 min. The results are expressed as optical density (OD) units. In the H1N1-EIA, samples were studied for the unspecific binding of antibodies to the uncoated wells (only PBS), but otherwise treated as antigen coated wells. The IgG binding to the uncoated wells was subtracted from the IgG binding to antigen coated wells. The intra-assay CV was 8.3% and inter-assay CV 11.5% for H1N1 antigen EIA.

H1N1 antigen suspension of Pandemrix or Arepanrix (final concentrations 1/250, 1/50 and 1/10) or Triton X and polysorbate 80 (the main detergents present in Pandemrix H1N1 antigen, at the final concentrations of 0.8, 4, 20 µg/ML) was added as a liquid-phase inhibitor in serum dilution buffer. The plasma samples with or without added inhibitor were incubated in room temperature for 2 hours and then studied in the EIA for H1N1 binding IgG-antibodies described above. The inhibition percent = 1-(OD value (uninhibited sample)/OD value (inhibited sample) multiplied 100.

#### Hemagglutination inhibition test for antibodies to H1N1

Hemagglutination inhibition (HI) test for antibodies against H1N1 influenza A/California/07/2009 vaccine strain and epidemic A/Finland/814/01 (H1N1) and A/Finland/715/00 (H3N2) virus strains was performed as previously described [24].

The neutralizing antibodies to Polio Sabin 1 virus were determined with a standard microneutralization assay as previously described [25].

#### Sandwich EIA for antibodies to Pandemrix derived H1N1 viral proteins

Polystyrene plates were coated with rabbit antibodies to HA, NA (anti-HA MAb (11055-RM05), anti-NA MAb (11058-R001) and anti-NP PAb (11675-RP01) all from Sino Biological, China), 1 µg/mL in PBS (100 µl/well), and kept overnight at +4°C 0.5% BSA-PBS was used for post-coating. After washing with PBS, Pandemrix antigen suspension (100 µl/well) was added and incubated in the antibody coated wells. After washing, mouse monoclonal antibodies to HA, NA or NP (anti-HA MAb (11055-MM08), anti-NA Mab (11058-MM07) or anti- NP MAb (11675-MM03) (1 µg/ml in 0.5% BSA-PBS) were added. In the EIA for IgG-antibodies to Pandemrix derived NP, plasma samples were diluted 1∶300 in 0.5% bovine serum albumin (BSA)-PBS. The binding to antibodies was detected with alkaline phosphatase conjugated anti-human IgG-antibody or anti-mouse IgG-antibody (both from Jackson ImmunoResearch, USA). After adding the substrate the color reaction was read.

#### EIA for antibodies to recombinant H1N1 viral proteins

Polystyrene plates were coated with recombinant HA from H1N1 influenza A/California/07/2009 vaccine strain or NP from A/Puerto Rico/8/1934 (H1N1) in PBS (both from Sino Biological, China), 0.1 µg/well. 0.5% BSA-PBS was used for post-coating, washing buffer was either 0.5% BSA-PBS or 0.5% BSA-0.05% PBS-polysorbate 80, and plasma samples were diluted 1∶100 in 0.5% BSA-PBS or 0.5% BSA-0.05%PBS-polysorbate 80. Alkaline phosphatase conjugated anti-human IgG-antibody (Jackson ImmunoResearch, USA) or anti-mouse IgG antibody (Jackson ImmunoResearch, USA) was used as a conjugate.

#### Genotyping of HLA DQB1*06∶02 risk allele of narcolepsy

HLA genotyping for the selected DQB1 and DQA1 alleles was performed using sequence-specific oligonucleotide hybridization as earlier described [26].

#### MES-PAGE analysis

All polyacrylamide gels were run by the NOVEX (Life Technologies, USA) High-resolution Bolt Mini Gel Tank System with ready-made Bolt 4–12% Bis-Tris Plus gradient gels under MES buffer conditions according to the instructions of the manufacturer. Gels were stained by PageSilver Silver Staining Kit from Fermentas Life Sciences (Thermo Scientific, USA) according to the instructions of the manufacturer. Pre-stained standard SeeBlue Plus 2 (NOVEX, Life Technologies, USA) was used for molecular weight calculations under reduced conditions. Non-**r**educing conditions allowed no molecular weight estimations. Recombinant Influenza A virus H1N1 nucleoprotein (NP) and HA (Sino Biologicals Inc., China) were used as control proteins.

#### Western blot analysis

All polyacrylamide gels were run by the NOVEX (Life Technologies, USA) High-resolution Bolt Mini Gel Tank System with ready-made Bolt 4–12% Bis-Tris Plus gradient gels under MES buffer conditions according to the instructions of the manufacturer. After the gel electrophoresis each gel was blotted by the iBlot Dry Blotting System with the pre-installed P0 program with the 10 minutes transfer selection according to the manufacturer instructions. Ready-made iBlot Gel Transfer Regular Nitrocellulose Stacks were used. Milk powder (4%) diluted in TBS-0.05% Tween20 was used for blocking, and mouse monoclonal antibodies against HA or NP (anti-HA MAb (11055-MM01) or anti- NP MAb (11675-MM03), Sino Biologicals) diluted at the concentration of 1 µg/mL in Tween- TBS was used for the identification of viral HA and NP. Alkaline phosphatase conjugated rabbit antibodies to mouse IgG was used as a secondary antibody.

#### Protein identification by mass spectrometry analysis

Excised gel bands of interest were washed and dehydrated with acetonitrile (ACN). Proteins were reduced with 20 mM dithiothreitol and incubated at 56°C for 30 min before alkylation with 55 mM iodoacetamide −0.1 M ammonium hydrogen carbonate (NH4HCO3) in the dark at room temperature for 15 minutes. After washing with 0.1 M NH4HCO3 and dehydration with ACN the gel pieces were rehydrated in 10 to 15 µl sequencing grade modified trypsin (Promega, USA) in 0.1 M NH4HCO3, to a final concentration of 0.015 µg/µl trypsin and incubated for digestion overnight at 37°C. Tryptic peptides were eluted from the gel pieces by incubating successively in 25 mM NH4HCO3 and then twice in 5% formic acid for 15 minutes at room temperature each. The resulting tryptic digest peptides were desalted using Zip Tip µC-18 reverse phase columns (Millipore, USA) and directly eluted with 50% ACN −0.1% trifluoroacetic acid (TFA) onto MALDI target plate. A saturated matrix solution of α-cyano-4-hydroxy cinnamic acid (CHCA) (Sigma, USA) in 33% ACN −0.1% TFA was added.

MALDI-TOF analyses were carried out with UltrafleXtreme 2000 Hz instrument (Bruker Daltonics, Bremen Germany) equipped with a SmartBeam II laser (355 nm), operated in positive and reflective modes. Typically, mass spectra were acquired by accumulating spectra of 10000 laser shots and up to 30000 for MS/MS spectra. External calibration was performed for molecular assignments using a peptide calibration standard (Bruker Daltonik GmbH, Leipzig, Germany). Protein identifications were performed by combining the files (PMF and few Lift spectra (MSMS) originating from the same gel band) and searching against open SwissProt database. ‘All entries’ option was selected in taxonomy field using Matrix Science’s Mascot Daemon (Matrix Science Ltd, UK). FlexAnalysis v3.4 and Biotools v3.2 softwares (Bruker Daltonics) were used to assign molecular isotopic masses to the peaks in the MS spectra and as search engine interface between mass list data transfer and the databases in Mascot server, respectively. The following parameters were set for the searches: 0.1 Da precursor tolerance and 0.5 or 1 Da MS/MS fragment tolerance for combined MS and MS/MS searches, fixed and variable modifications were considered (carbamidomethylated cysteine and oxidized methionine, respectively), one trypsin missed cleavage was allowed.

#### Isolation of lipid-protein micelles by sequential ultracentifugation

Isolation of lipid-protein micelles was performed by sequential ultracentrifugation using Table-Top ultracentrifuge (Beckmann Optima TL, USA) and KBr for density adjustments [27], [28]. Pandemrix vaccine sample (2 mL) mL) was first adjusted to the density (d) of 1.006 g/mL and the centrifuge tube filled with a d = 1.006 g/mL KBr solution (1 mL) to the total volume of 3 mL. The samples were centrifuged at +5C for 2 hr at the speed 100 000 rpm (corresponding relative centrifugal field 500 000 × g). After centrifugation the top 1 mL fraction was recovered and the bottom infranatant fraction was adjusted to the density of 1.063 g/mL using solid KBr, filled to 3 mL with d = 1.063 g/mL KBr solution and centrifuged (+5C, 3 hr, 100 000 rpm). The top 1 mL fraction was recovered. To get the final fraction corresponding to the density of human HDL particles the bottom fraction was adjusted with solid KBr to the density of 1.21 g/mL, the vials filled with KBr 1.21 g/mL density solution and then centrifuged (+5C, 18 hrs, 100 000 rpm). The top 1 mL fraction was again aspirated and all the isolated density fractions, i.e. D<1.006 g/mL, D = 1.006–1.063 g/mL and D = 1.063–1.21 g/mL, D>1.21 g/mL, and aliquots from all the bottom infranatant fractions were dialyzed against phosphate-buffered saline (PBS, pH 7.4) before use.

The isolated density fractions were analysed for the presence of viral HA, NA and NP with dot-blot method, in which the 5 µl of fractions were pipetted to the nitrocellulose membrane and after residual coating with dried milk the presence of proteins were detected with antibodies to HA, NA and NP (11055-MM01,11058-MM07or 11675-MM03, respectively, from Sino Biologicals). Alkaline phosphatase conjugated rabbit antibodies to mouse IgG was used as a secondary antibody.

### Statistical methods

The distribution of antibodies in the study groups was not normal and thus the statistical analyses were performed using OD values and antibody titers after logarithmic transformation. The groups were then compared by using unpaired t-test. Percent inhibition values caused by Pandemrix and Arepanrix H1N1 antigen suspension were compared with paired t-test after logarithmic transformation.

## Results

### Increased IgG-antibody response to H1N1 viral antigen of Pandemrix in narcolepsy

**Figure 1:**
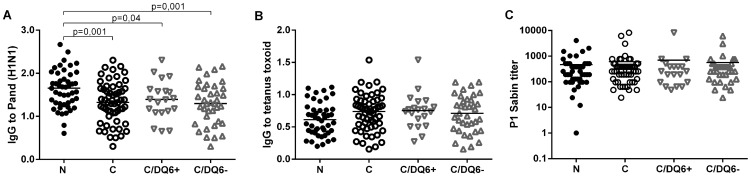
The IgG-antibody levels to H1N1 antigen suspension of Pandemrix (A), tetanus toxoid (B) and neutralizing antibody levels to Polio Sabin vaccine virus (C) in 47 children with narcolepsy (N) and 57 healthy vaccinated children with and without HLA DQB1*06∶02 risk allele of narcolepsy (C/DQ6+ and C/DQ6−). Antibodies to Pandemrix H1N1 antigen and tetanus toxoid were determined by EIA and the results expressed as optical density units (y-axis).

We studied the IgG-antibody response to H1N1 viral antigen suspension using EIA method, and observed that children with narcolepsy showed higher levels of IgG-antibodies binding to the Pandemrix H1N1 antigen suspension than did vaccinated healthy children from the general population (Fig. 1A ). This difference was still evident when healthy children with HLA DQB1*06∶02 risk allele of narcolepsy were compared to the children with narcolepsy (Fig. 1 A). To control for the possible hyper-reactivity of antibody response to vaccine antigens in general, we studied antibodies to tetanus toxoid with EIA and neutralizing antibodies to polio Sabin virus. In these control tests, no differences in antibody levels were found between children with narcolepsy and healthy children (Fig. 1B–C).

### Antigenic difference between H1N1 antigen suspension of Pandmerix and Arepanrix

**Figure 2:**
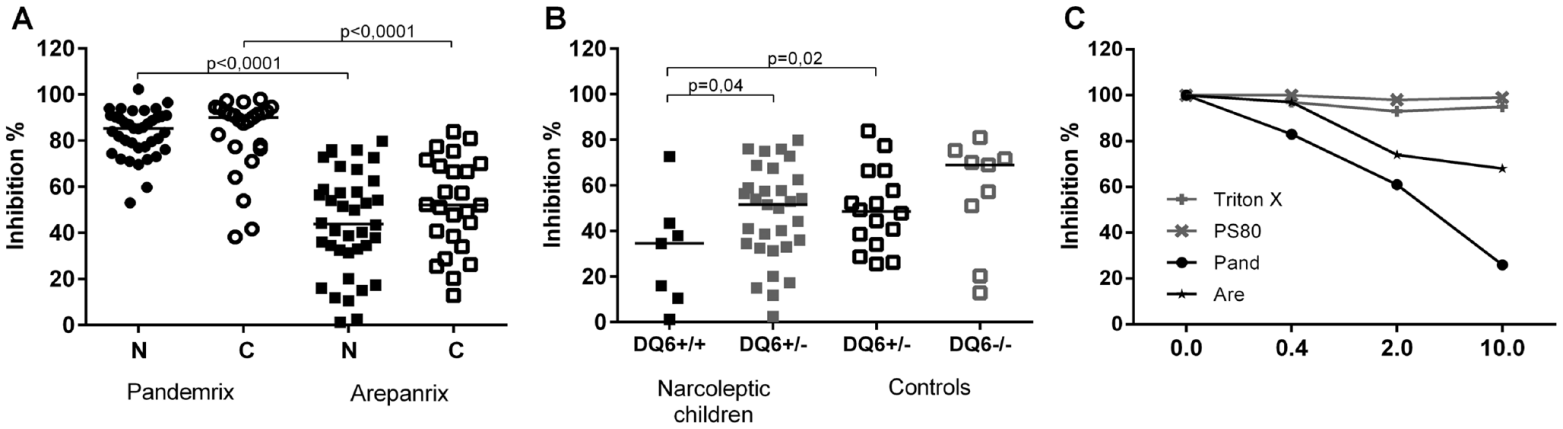
The *in vitro* inhibition of IgG-antibodies binding to H1N1 antigen of Pandemrix is evaluated using H1N1 antigen suspension of Pandemrix, Arepanrix or detergents present in Pandemrix as liquid-phase inhibitors pre-incubated with plasma samples from study subjects. A. The inhibition of IgG-antibodies binding to solid-phase bound H1N1 antigen of Pandemrix (% of inhibition in y-axis) is lower when Arepanrix antigen (1/10) is used as inhibitor compared to Pandemrix (1/10) as an inhibitor both in children with narcolepsy (N; n = 47) and in healthy vaccinated children (C; n = 57). **B.** The inhibition of IgG-antibodies binding to solid-phase bound H1N1 antigen of Pandemrix (% of inhibition in y-axis) with Arepanrix antigen(1/10) is weaker in the children with narcolepsy who are homozygotic for HLA DQB1*06∶02 risk allele than in the children with narcolepsy or healthy children who are heterozygotic for HLA DQB1*06∶02 risk allele of narcolepsy. **C.** Dose-dependent inhibition of IgG-antibodies binding to solid-phase bound H1N1 antigen of Pandemrix (% of inhibition in y-axis) using as a liquid phase inhibitor H1N1 antigen suspension of Arepanrix or Pandemrix at the final dilutions of 1/250, 1/50 and 1/10 (0.4, 2, 10 on x-axis) or the detergents present in the H1N1 antigen suspension of Pandemrix, Triton X and polysorbate 80, at the final concentrations 0.8, 4, and 20 µg/mL (0.4, 2, 10 on axis). The inhibition curves represent mean value of the % of inhibition in plasma samples from 3 children with narcolepsy.

To study the possible antigenic differences between the Pandemrix and Arepanrix H1N1 antigen preparations, we performed inhibition experiments in which the binding of plasma IgG-antibodies to H1N1 antigen of Pandemrix was inhibited with either H1N1 antigen of Pandemrix itself or H1N1 antigen of Arepanrix. The inhibition of IgG-antibodies to Pandemrix H1N1 viral antigen was significantly weaker with the Arepanrix H1N1 antigen than with the Pandemrix H1N1 antigen in both children with narcolepsy and healthy, vaccinated children (P<0.0001, Fig. 2A), which indicates antigenic differences between the viral components of Arepanrix and Pandemrix. In the group of children with narcolepsy who were homozygotic for HLA DQB1*06∶02 risk allele of narcolepsy, the inhibition of IgG-antibodies with Arepanrix was weaker than in the children with narcolepsy or healthy children who were heterozygotic for HLA DQB1* 06∶02 (Fig. 2 B, P = 0.04 and P = 0.02, respectively), which suggests that the antibody response to the antigenic epitopes that differ between Pandemrix and Arepanrix are regulated by HLA DQB1* 06∶02 restricted T-cells. The detergents present in Pandemrix H1N1 antigen did not inhibit the binding of antibodies to H1N1 antigen of Pandemrix as shown in Fig. 2 C, where a dose dependent inhibition of the antibodies binding to the Pandemrix H1N1 antigen is demonstrated with Pandemrix and Arepanrix H1N1 antigen and with detergents used in the manufacturing of Pandemrix.

### Protein composition of Pandemrix and Arepanrix H1N1 viral suspension differ

**Figure 3:**
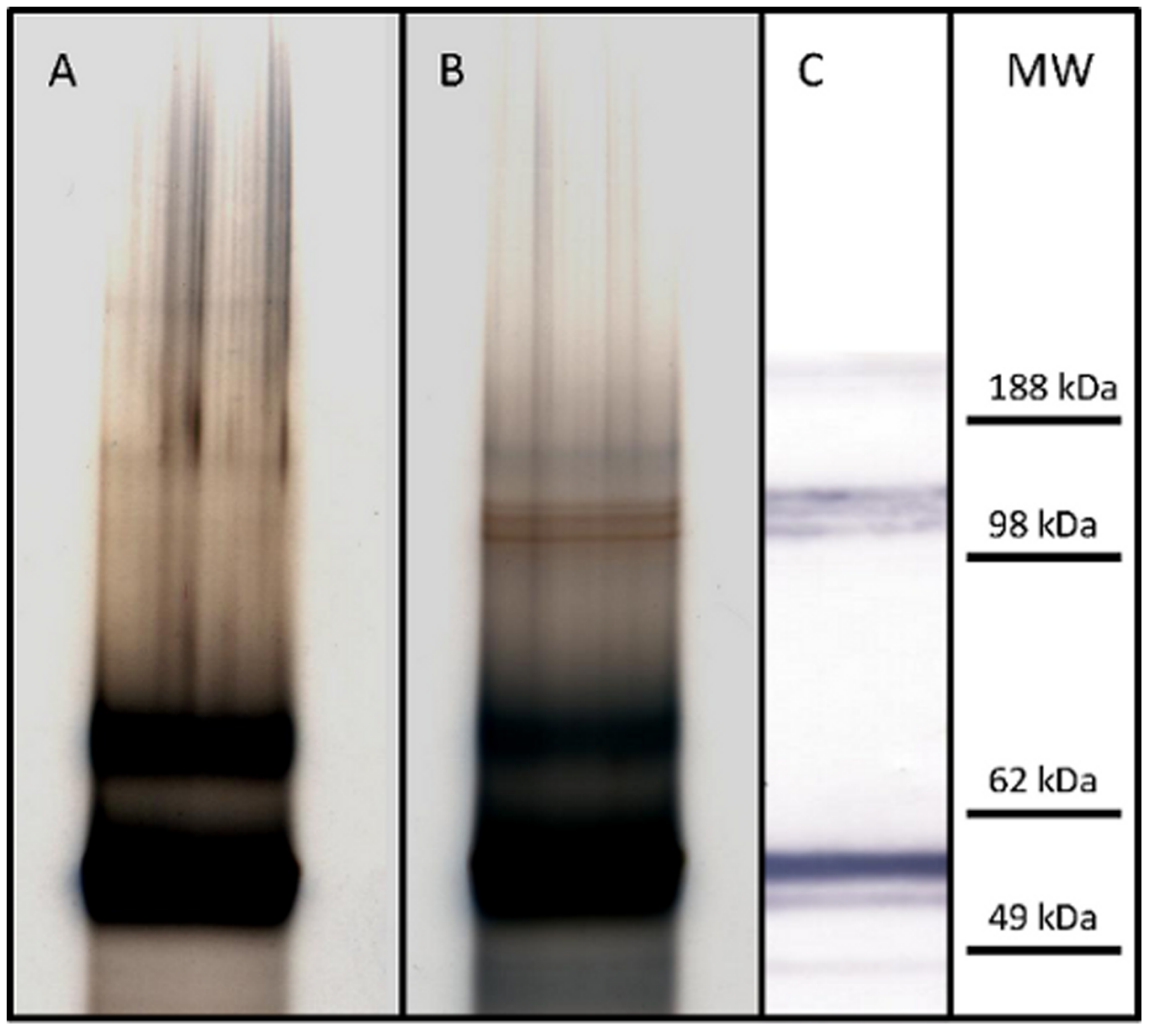
High-resolution MES-PAGE analysis of Arepanrix (lane A) and Pandemrix (lane B) H1N1 antigen suspensions under reducing conditions, and immunostaining of the separated proteins in Pandemrix by western blotting using anti-NP antibody (C). A set of sharp bands at approximately 120 kDa molecular weight could be seen in Pandemrix (B) but not in Arepanrix sample (A). These bands in Pandemrix were identified by mass spectrometry to be polymeric forms of the influenza virus nucleoprotein (NP) and by western blotting with anti-NP antibody (C).

In order to study the possible differences in the protein content of H1N1 viral antigen suspensions, we performed High-resolution MES-polyacrylamide gel electrophoresis analysis. We analyzed both Pandemrix and Arepanrix H1N1 antigen suspensions in MES gels under reducing conditions and noticed that Pandemrix repeatedly showed a set of sharp bands at approximately 120 kDa molecular weight (reducing conditions) which could not be seen in the Arepanrix sample (Fig. 3). These bands in Pandemrix were identified by mass spectrometry to be polymeric forms of the influenza virus nucleoprotein (NP).

**Figure 4:**
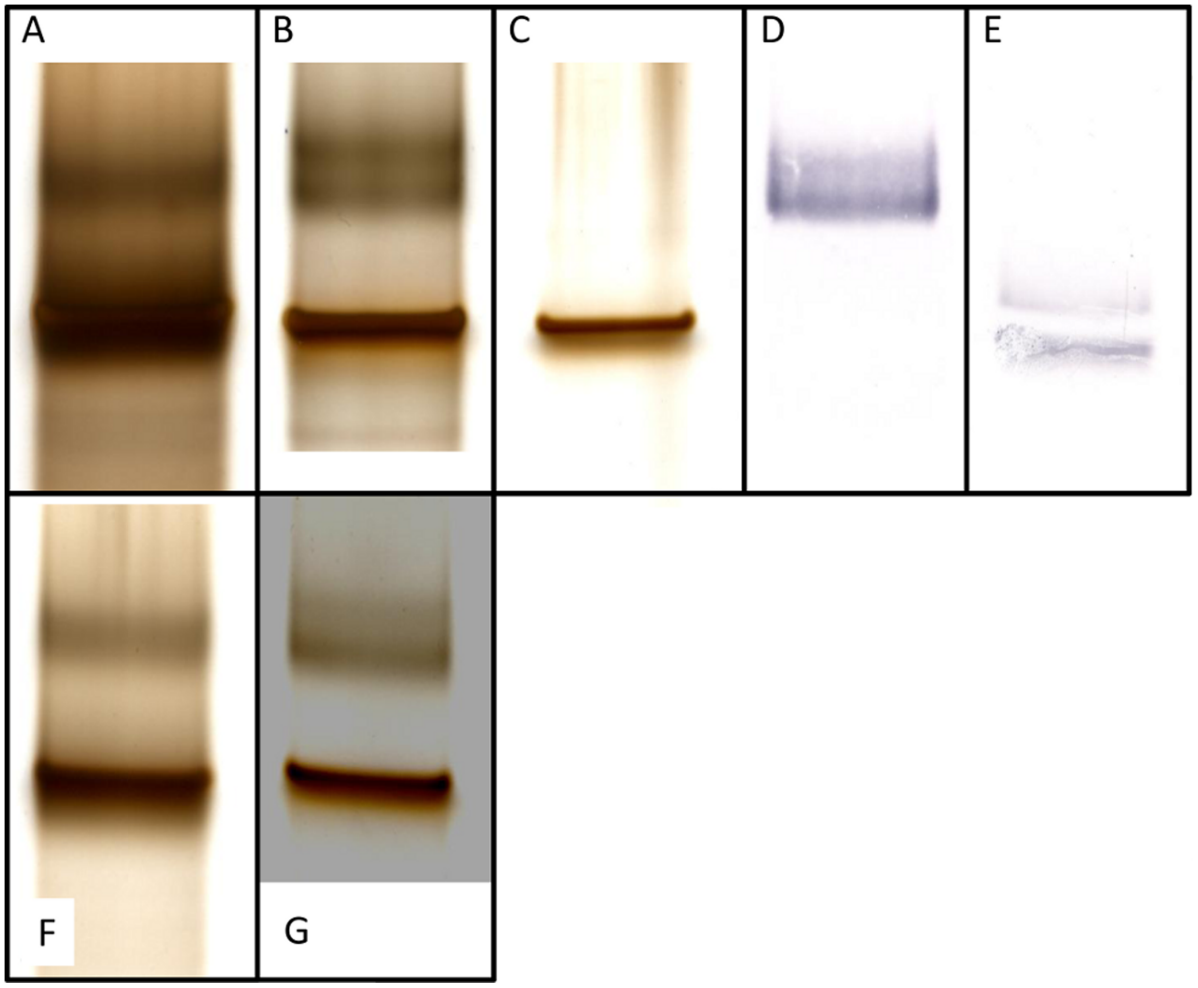
High-resolution MES-PAGE analysis of Pandemrix and Arepandrix vaccines under reducing and non-reducing conditions. Pandemrix H1N1 antigen suspension run under reducing conditions (A) with a major band including a mixture of HA and NP, and under non-reducing conditions (B) with HA shown in the upper area and NP at its original place identical to RF position of the reducing gel (A). Recombinant NP from H1N1/A/Puerto Rico 1934 (Sino Biologicals Inc., China) alone run under reducing conditions (C). HA stained with monoclonal mouse anti-HA antibody (Sino Biologicals Inc., China) using western blotting of the separated proteins of Pandemrix H1N1 antigen suspension under non-reducing conditions (D) and under reducing conditions (E). Arepanrix H1N1 antigen suspension run under reducing conditions (F) with a major band including a mixture of HA and NP (F), and run under non-reducing conditions (G) with HA shown in the upper area and NP at its original place identical to RF position of the reducing gel (F).

We then performed an additional analysis of the same samples under non-reducing conditions, allowing the separation of HA and NP from each other (Fig. 4). HA and NP share almost identical molecular weights and thus co-migrate under reducing conditions in a polyacrylamide gel practically forming one band, but are clearly separated under non-denaturing conditions. MES-PAGE analysis under non-reducing conditions verified a relative large amount of NP in the both Pandemrix and Arepanrix antigen suspensions as compared to the HA amount in the same samples, but as shown in Fig. 3, the polymeric NP was basically undetectable in Arepanrix in MES-PAGE.

### Western blot analysis of viral proteins in H1N1 antigen suspension of Pandemrix

**Figure 5:**
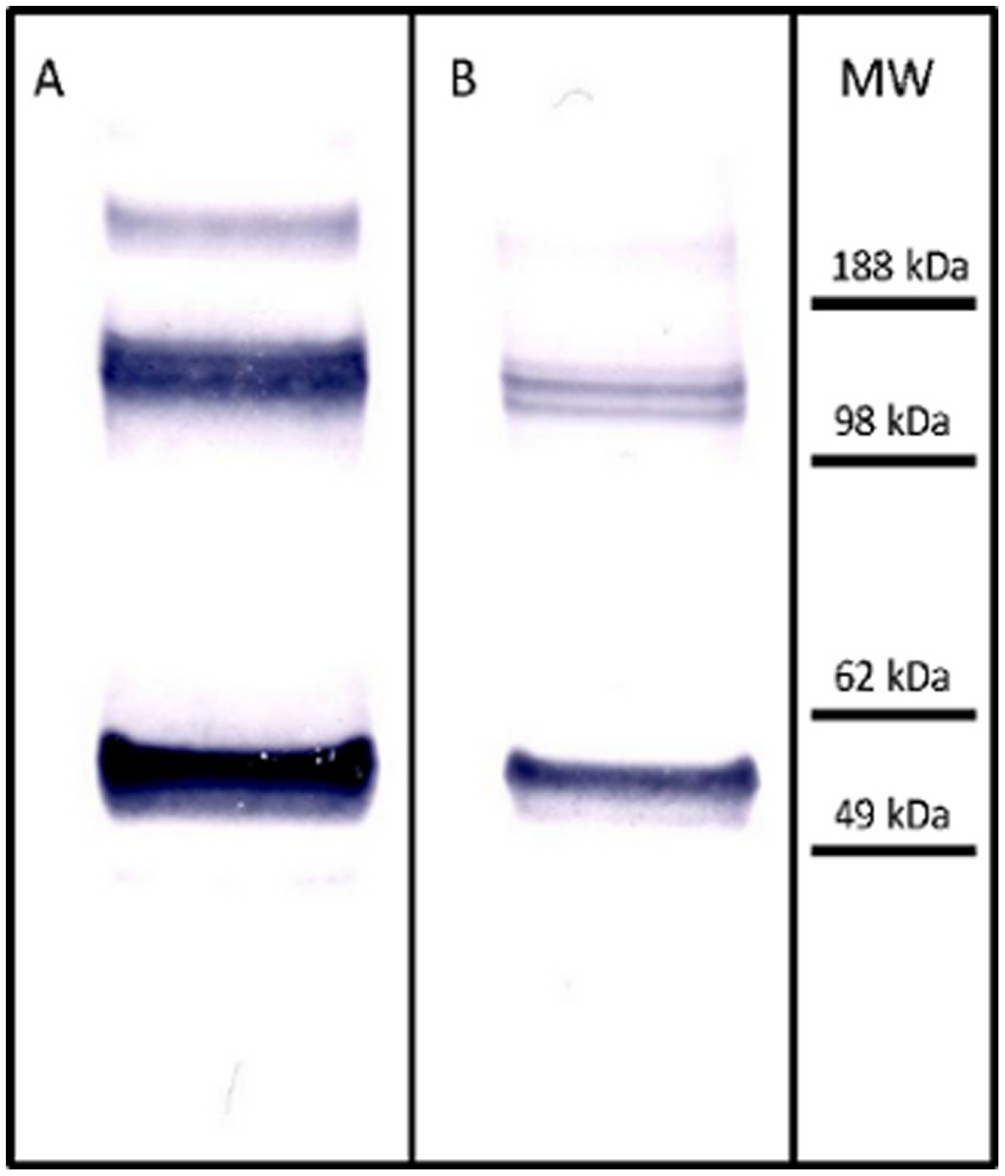
Western-blot analysis of the presence of NP in Pandemrix and Arepanrix H1N1 antigen suspension. Pandemrix and Arepanrix H1N1 antigen suspensions were run under reducing conditions and NP stained with monoclonal mouse NP-antibody (Sino Biologicals Inc., China).

In order to verify the identity of the NP polymer bands, we analyzed Pandemrix and Arepanrix samples parallel by western blotting using anti-NP antibodies. Each polymer was clearly identified by the NP antibody as shown in Fig. 5. According to this data Pandemrix seems to have different content of polymerized NP proteins and a large amount of NP present in comparison to Arepanrix (Fig. 5).

In order to confirm the co-migration of NP and HA under reducing gel conditions, we performed a western blot analysis of the viral HA in both samples under reducing and non-reducing conditions. As shown in Fig. 4D and 4E, HA shifts clearly from its original band position in the reducing gel as compared to the corresponding position in the non-reducing gel. Unmasking NP from HA under non-reducing conditions allowed us to verify the large amount of NP present in both samples. Dual bands seen of both NP and HA at the 60 kDa molecular weight marker level under reducing conditions (Fig. 3C and 4E) indicate that each protein masks the epitope response from the other due to the partial overlap of the bands.

### Increased IgG-antibody response in narcolepsy to H1N1 viral proteins of Pandemrix

**Figure 6:**
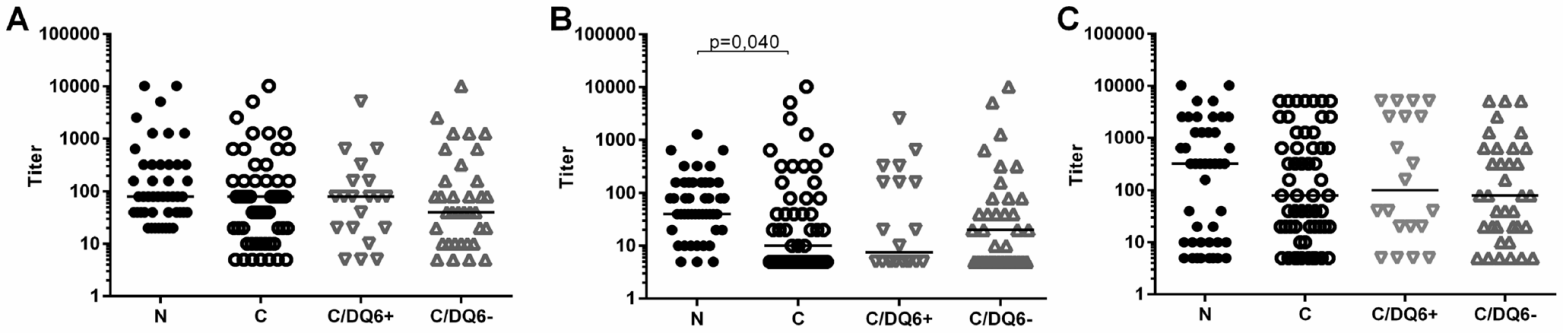
Antibody levels to HA as antibody titers determined with the hemagglutination inhibition (HI) test. Antibody titers against influenza A/California/07/2009 vaccine strain (A), epidemic A/Finland/814/01 (H1N1) virus strain (B) and influenza A/Finland/715/00 (H3N2) virus strain (C) in plasma samples from 47 children with narcolepsy and in 57 healthy vaccinated children.

**Figure 7:**
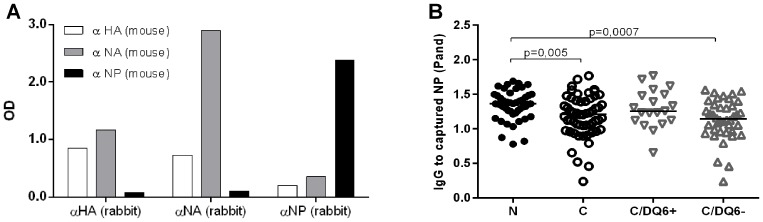
A sandwich EIA for the detection of IgG-antibodies to H1N1 proteins captured from H1N1 antigen of Pandemrix with solid-phase bound antibodies to HA, NA and NP. A. Capture of H1N1 viral proteins from Pandemrix antigen in a sandwich EIA revealed that HA and NA are found in a complex in the Pandemrix H1N1 antigen, whereas NP does not complex with HA or NA. H1N1 viral proteins from Pandemrix vaccine were captured with rabbit monoclonal anti-HA, anti-NA and anti-NP antibodies bound to EIA plate and mouse monoclonal anti-HA, anti-NA and anti-NP antibodies were used as the detection antibodies. B. IgG-antibodies binding to Pandemrix derived NP were higher in children with narcolepsy (N; n = 47) than in vaccinated healthy children(C; n = 57) when studied with sandwich EIA using anti-NP antibody to capture NP from Pandemrix H1N1 antigen suspension. The children with narcolepsy (N) had higher levels of IgG-antibodies to Pandemrix derived NP than the healthy, vaccinated children without HLA DQB1*06∶02 risk allele (C/DQ6−; n = 37), whereas no difference was seen in comparison with the healthy, vaccinated children with HLA DQB1*06∶02 risk allele (C/DQ6+; n = 20).

We then compared the IgG-antibody reactivity in children with narcolepsy and in healthy children against H1N1 viral proteins; HA and NA the major immunogens in influenza vaccines; and NP, a viral antigen found in a significant amount in Pandemrix according to our analyses. We first measured antibodies to HA using the HI test, in which antibody response to native viral HA is detected. We found a trend for higher titers of antibodies against influenza A/California/07/2009 vaccine strain and epidemic A/Finland/814/01 (H1N1) whereas no differences were seen in HI titers against or A/Finland/715/00 (H3N2) virus strains (Fig. 6). In order to evaluate the antigenic moieties in the vaccine derived viral proteins HA, NP, and NA, we developed a sandwich EIA, in which the protein from the H1N1 viral antigen suspension of Pandemrix is captured with a specific monoclonal antibody coated on the polystyrene wells. These coated, antigen containing plates were then used to determine plasma IgG binding to vaccine derived protein. When the specificity of the sandwich EIA for HA, NP, and NA was analyzed using monoclonal antibodies to viral proteins as detection antibodies, we found that detection antibody to NA bound to HA captured from Pandemrix, and *vice versa*, which suggests the presence of HA-NA complexes in H1N1 antigen suspension of Pandemrix (Fig. 7 A). The sandwich EIA for NP captured from Pandemrix appeared to be antigen specific (Fig. 7 A). We found that the binding of antibodies to NP captured from Pandemrix was enhanced in children with narcolepsy when compared to healthy vaccinated children (Fig. 7 B). Due to the complex formation of HA and NA, the specific antibodies against Pandemrix derived HA or NA could not be determined.

### Lipid-protein micelles in H1N1 antigen of Pandemrix

**Figure 8:**
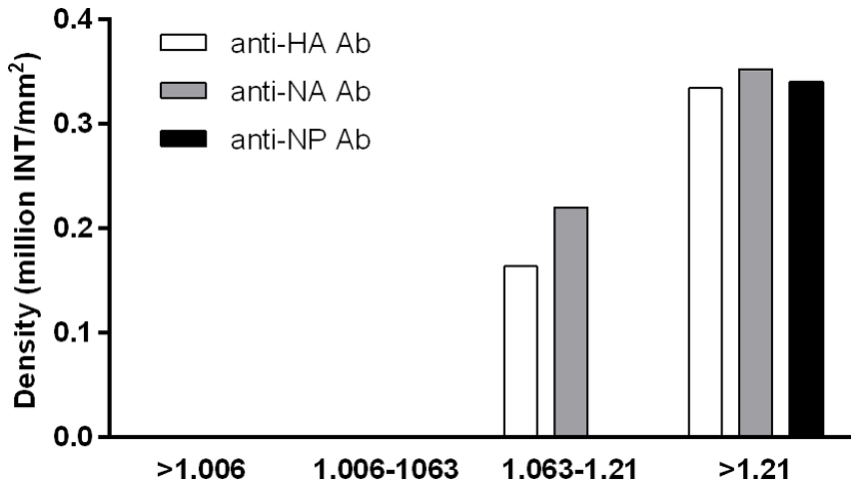
Dot blot detection of viral proteins with mouse monoclonal anti-HA, anti-NA and anti-NP antibodies (Sino Biologicals Inc., China) in the density fractions isolated with gradient ultracentrifugation from Pandemrix H1N1 antigen suspension. HA and NA was detected as lipid-protein micelles in the density fraction corresponding density between 1.063–1.21 g/mL, and soluble HA, NA and NP were detected in the density fraction above 1.21 g/mL.

Because polysorbate 80 and Triton X, the non-ionic lipid detergents used for the manufacturing process of the Pandemrix H1N1 antigen suspension, are able to form micelles with proteins and lipids, and viral proteins could be in a complex due to micelle formation, we studied whether the viral proteins were incorporated in lower density lipid micelles in H1N1 antigen suspension. We separated micelles with different lipid-protein composition from Pandemrix H1N1 antigen suspension using a sequential density ultracentrifugation method validated for the isolation of human HDL, LDL and VLDL lipoprotein classes. We identified HA, NA and NP with specific antibodies in the density fractions indicating the presence of lipid-protein micelles (Fig. 8).

### Antibody response to H1N1 viral nucleoprotein is regulated by HLA DQB1*06∶02

**Figure 9:**
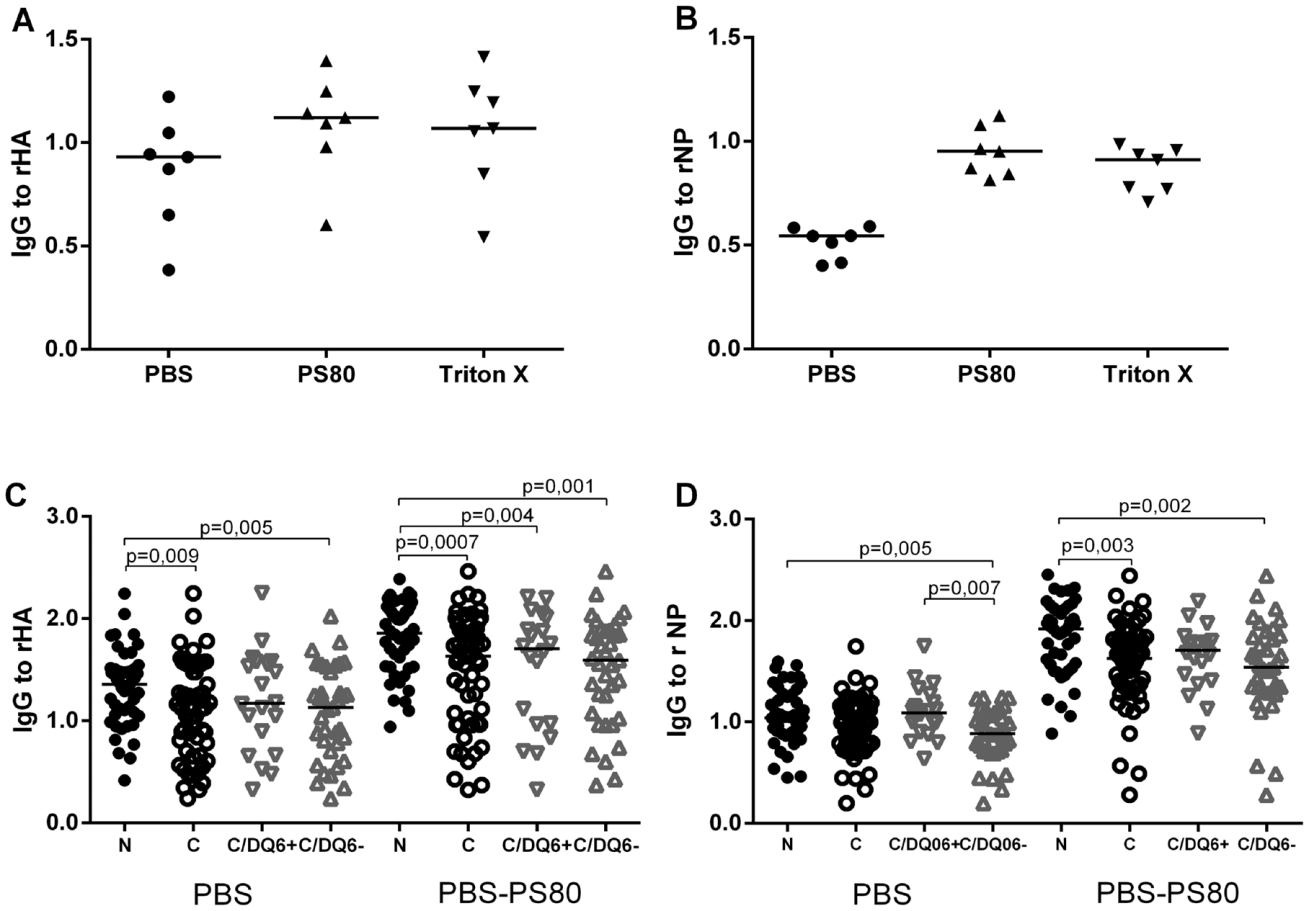
Antibodies to untreated and detergent treated recombinant viral proteins studied by EIA. IgG-antibodies against untreated recombinant viral proteins, rHA (A) and rNP (B), and against rHA and rNP exposed to non-ionic detergents, Triton X and polysorbate 80 (PS80), used in the manufacturing process of Pandemrix H1N1 antigen, but not Arepanrix, in six plasma samples from vaccinated children with narcolepsy C. Children with narcolepsy (N; n = 47) had higher levels of IgG-antibodies to polysorbate 80 treated recombinant NP (PS80) but not to untreated NP (PBS) than healthy vaccinated children (C; n = 57). Children with narcolepsy who all carry HLA DQB1*06∶02 allele (N; n = 47) and healthy vaccinated children with HLA DQB1*06∶02 allele (C/DQ6+; n = 20)) had higher levels of antibodies to untreated NP in comparison to the children without HLA DQB1*06∶02 allele (C/DQ6−; n = 37). D. Children with narcolepsy (N; n = 47) showed higher levels of IgG-antibodies to untreated recombinant HA (PBS) and polysorbate 80 treated HA (PS80) than healthy vaccinated children (C; n = 57). No difference was found in the antibody levels to treated or untreated HA between healthy children with or without HLA DQB1*06∶02 allele (C/DQ6+; n = 20 and C/DQ6−; n = 37).

Next, we used recombinant HA and NP (rHA and rNP) in EIA to explore the possible differences in the antibody reactivity to the H1N1 viral proteins of interest in children with narcolepsy and healthy children. Because we found increased antibodies to Pandemrix derived NP in narcolepsy, which was exposed to detergents and associated with lipid micelles, we also wanted to address the possibility that the antigenic properties of the viral antigens could be modified by the detergents used in the manufacturing process, namely polysorbate 80 and Triton X100. Both non-ionic detergents modified the antigenic properties of viral antigens, particularly rNP, and an enhanced binding of IgG antibodies to the detergent exposed rNP was seen in children with narcolepsy (Fig. 9 A and B). We therefore used only polysorbate 80 in subsequent experiments on detergent treated antigens. We then compared the binding of IgG-antibodies to rNP with and without exposure to polysorbate 80 in children with narcolepsy and healthy children. We found that the children with narcolepsy showed higher levels of antibodies to detergent modified rNP but not to untreated when compared to healthy vaccinated children (Fig. 9C). This suggests that IgG-antibodies from children with narcolepsy differently recognize detergent modified virus antigen moieties in rNP than IgG-antibodies from healthy vaccinated children. Healthy children with DQB1*06∶02 risk allele showed higher levels of antibodies to rNP than children without DQB1*06∶02 allele, but interestingly the levels of antibodies to rNP were higher in the children with narcolepsy even when compared to the children with DQB1*06∶02 risk allele. The antibody levels to untreated rHA and detergent treated rHA were higher in children with narcolepsy than in healthy vaccinated children, but no association of antibodies to HA was found with the presence of DQB1*06∶02 risk allele (Fig. 9D).

The antibodies to detergent treated and untreated rHA showed a strong correlation with antibodies to H1N1 antigen suspension of Pandemrix in both children with narcolepsy and healthy children as expected (R = 0.726 for detergent untreated rHA and R = 0.628 for detergent treated rHA; p<0.0001 for both; R = 0.762 and R = 0.714; p<0.0001, respectively). Instead, the antibodies to rNP correlated with antibodies to H1N1 antigen of Pandemrix only in children with narcolepsy (R = 0.356; p = 0.013 for untreated rNP and R = 0.417;p = 0.003 for detergent treated rNP), but not in the healthy children (R = 0.08; p = 0.529 for untreated rNP and R = 0.137; p = 0.308 for detergent treated rNP).

**Figure 10:**
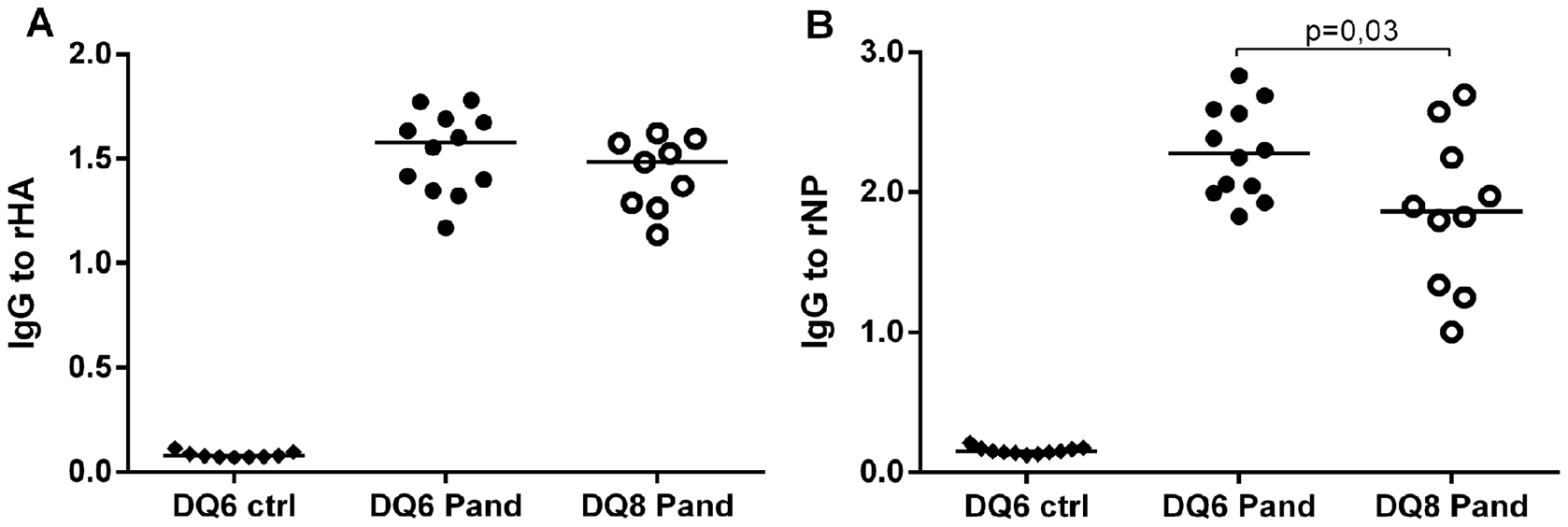
IgG-antibody levels to detergent treated recombinant HA (A) and NP (B) in the Pandemrix immunized mice transgenic for HLA DQB1*06∶02 (DQ6 Pand) or HLA DQB1*03∶02 (DQ8 Pand) and in the HLA DQB1*06∶02 transgenic mice immunized with PBS (DQ6 ctrl). IgG-antibodies to rNP are higher in the immunized HLA DQB1*06∶02 (DQ6 Pand) than in the immunized HLA DQB1*03∶02 transgenic mice (DQ8 Pand), whereas no difference is seen in the antibody levels to rHA in the EIA.

We then immunized with Pandemrix NOD mice transgenic for the narcolepsy related human DQB1*06∶02 allele or the type 1 diabetes related DQB1*03∶02 allele and studied the antibody response to detergent exposed rHA and rNP. We observed that IgG-antibodies induced by Pandemrix vaccination to rNP were higher in the HLA DQB1*06∶02 transgenic mice when compared HLA DQB1*03∶02 transgenic mice, whereas no difference was seen in the antibodies to rHA (Fig. 10).

## Discussion

The aim of the current study was to explore the possible antigenic differences between two AS03 adjuvanted H1N1 vaccines, Pandemrix and Arepanrix, in order to elucidate the mechanisms behind Pandemrix-associated narcolepsy. Importantly, we provide the first evidence of significant antigenic differences between the Pandemrix and Arepanrix H1N1 antigen suspensions. Based on these immunological findings and the epidemiological observations of Pandemrix-associated narcolepsy, we suggest that the H1N1 viral antigen suspension of Pandemrix is in a key role in the triggering vaccine-induced narcolepsy. The presence of an antigen-specific trigger of narcolepsy in the Pandemrix vaccine is also suggested by the observations that no increased risk of other autoimmune diseases has been observed in association with Pandemrix vaccination, only narcolepsy [8].

While these two AS03 adjuvanted H1N1 vaccines Pandemrix and Arepanrix were produced according to different methods of antigen purification, they were considered to be comparable [19]. However, using both immunological and proteomics approach we found antigenic differences between the two preparations. We demonstrated that the viral protein NP was present in higher amount in Pandemrix than in Arepanrix, particularly structurally altered NP. Given that Pandemrix vaccine antigen was produced under different conditions than Arepanrix, the purification process of viral proteins has likely resulted in the differential enrichment of NP. Moreover, it seems that these production differences have led to a yet unknown cascade of detergent influenced protein polymerization. Polymeric proteins are known to be more immunogenic than monomeric proteins, and polymerization may also result in the changes in the antigenic epitopes in NP as reported earlier [29].

Our immunological studies revealed that children with narcolepsy have higher levels of IgG-antibodies to Pandemrix derived NP and *in vitro* detergent treated recombinant NP than healthy children. These differences in the antibody response could result in differences in the presentation of viral antigens by B-cells or other APCs that uptake antibody-bound antigens, which are then processed and presented in the complex with HLA class II molecules, such as DQB1*06∶02. The crystal structures of membrane proteins have revealed how hydrophobic parts of detergents tend to bind to the hydrophobic regions of proteins [30]. Hermeling et al. observed that erythropoietin alpha formulations contained small quantities of polysorbate 80 micelle-associated etythropoietin protein, which was suggested to increase immunogenicity of erythropoietin [31].

We found that the high antibody response to NP was associated with the DQB1*06∶02 allele in the children and also in transgenic mice expressing human DQB1*06∶02. No development of the symptoms of narcolepsy was seen in the mice transgenic for human DQB1*06∶02, which could be related to the differences in TCR-repertoire and structure of autoantigens between mice and humans. Our findings indicate however that the DQB1*06∶02 risk allele of narcolepsy is an important regulator of immune response to NP, and the children with narcolepsy show altered antibody reactivity to Pandemrix derived NP when compared to healthy vaccinated children. The impaired inhibition of antibody reactivity to Pandemrix H1N1 antigen with Arepanrix antigen in the individuals who were homozygotic for DQB1*06∶02 is likely explained by the differences in the antigenicity of polymeric NP in Pandemrix. The association of an enhanced antibody response to NP with narcolepsy and the disease related HLA risk allele together with the findings of high amounts of NP and its polymeric forms in Pandemrix, provide a clue for the role of NP in the development of narcolepsy. Our findings here do not provide a mechanistic link between the immune response to NP and narcolepsy, however. Given a role of DQB1*06∶02 allele in the presentation of antigenic epitopes to CD4 T-cells, it is urgent that NP derived DQB1*06∶02 dependent epitopes are screened. Because of limited sample availability from children, the protein composition of Pandemrix and Arepanrix had to be determined before proceeding to T-cell epitope screens. This proposed screen could reveal the environmental driving antigen of narcolepsy related autoimmunity.

If Pandemrix contained an antigen-specific trigger of narcolepsy, as suggested by the epidemiological and immunological evidence discussed above, the increased risk of narcolepsy in the vaccinated individuals may be seen years after vaccination. On the other hand, it also explains why in some individuals such a rapid development of narcolepsy was seen after Pandemrix vaccination [32]; in some cases the underlying narcolepsy related autoimmunity was specifically re-activated by Pandemrix.

The association of H1N1 virus infection with increased incidence of narcolepsy has been reported in China [16], [17]. Our serological studies performed in the same series of patients as studied here do not provide evidence for the role of H1N1 infection as a contributing factor for the increase of narcolepsy in children and adolescents observed in 2009–2010 [33]. The ecological studies in South Korea do not either support the association of H1N1 infection and narcolepsy [34]. It is of interest that H1N1 vaccines contain NP, which originates from H1N1 Puerto Rico 1934 strain and not from the pandemic H1N1 influenza strain.

A role of H1N1 virus derived HA in narcolepsy was suggested based on the molecular mimicry of DQB1*06∶02 binding epitopes between HA and hypocretin peptides [18], but this report was later retracted due to the lack of reproducibility of the findings. Here we show that the children with narcolepsy have enhanced antibody reactivity to recombinant HA. Increased antibody levels to H1N1 viral HA were reported also in Swedish children with narcolepsy when measured with a radio-immunoprecipitation assay using *in vitro* coupled transcription translation HA [35]. However, no significant difference was seen in the antibody reactivity of the healthy children and children with narcolepsy when HA antibody titers were studied in the HI test using the H1N1 pandemic strain (A/California/7/2009), the strain used in the Pandemrix vaccine. The role of HA as a trigger of narcolepsy is further challenged by the lack of the epidemiological evidence associating narcolepsy with other H1N1 vaccines containing also HA as the major immunogen. This is despite all pandemic and seasonal flu-vaccines administered after 2009 have contained immunogenic HA from H1N1 A/California/7/2009 virus, including Arepanrix, which contained the same adjuvant as Pandemrix, i.e., AS03.

The market authorization holder of both Arepanrix and Pandemrix, GSK, sponsored epidemiological research on narcolepsy in association with Arepanrix for a more robust assessment of the association than the studies that had been performed in Europe for Pandemrix [19]. In order to determine antigen equivalence of the two preparations they used HI test [19]. Our observations here imply that the HI test is too limited in scope to reveal quantitative and qualitative differences in the myriad immunogens found in these H1N1 vaccine preparations.

No prospective, pre-vaccination samples had been taken before the Pandemrix vaccination, thus it was not possible to evaluate the induction of antibodies to viral proteins by Pandemrix vaccination. Other limitations of our study are related to the availability of reagents. We received only ten vials of Arepanrix used in Canada during pandemic and were therefore unable to perform all immunological experiments in parallel with both Arepanrix and Pandemrix. Additionally, it would have been interesting to compare the immune response between Arepanrix and Pandemrix vaccinated children with and without risk genotype of narcolepsy, but samples from Arepanrix vaccinated children were not available.

Our results show that children who developed narcolepsy after Pandemrix vaccination developed altered immune response to Pandemrix. Given that antibody response to Pandemrix derived NP was increased in narcolepsy and the DQB1*06∶02 risk allele of narcolepsy appeared to regulate anti-NP immune rseponse, our data suggest that anti-NP immunity could be a link between Pandemrix and narcolepsy related autoimmunity, but our studies here do not provide data on the mechanisms. Importantly, we provide the first evidence of significant antigenic differences between the Pandemrix and Arepanrix H1N1 antigen suspensions produced by different methods, particularly the presence of high amounts of structurally altered NP in Pandemrix. We also demonstrated detergent-induced modifications of viral NP, which are readily recognized by antibodies from children with narcolepsy. The immunologically important differences in the H1N1 antigens of Arepanrix and Pandemrix could explain the difference observed in the vaccine-attributable risk of narcolepsy in these two AS03 adjuvanted H1N1 vaccines. The role of AS03 may have been indispensable as a booster of the immune response triggering narcolepsy or as an inducer of strong cross-reactive and polyfunctional CD4 T-cell responses against viral antigens as earlier shown by the market authorization holder [36]. Our findings shift the focus away from the adjuvant alone and instead suggest the H1N1 viral antigens, and particularly viral NP, as the prime suspect in the search for the trigger of vaccine-induced narcolepsy.
